# Effects of aerobic and resistance exercise on cardiac remodelling and skeletal muscle oxidative stress of infarcted rats

**DOI:** 10.1111/jcmm.15191

**Published:** 2020-04-02

**Authors:** Mariana J. Gomes, Luana U. Pagan, Aline R. R. Lima, David R. A. Reyes, Paula F. Martinez, Felipe C. Damatto, Thierres H. D. Pontes, Eder A. Rodrigues, Lidiane M. Souza, Ingrid F. Tosta, Ana A. H. Fernandes, Leonardo A. M. Zornoff, Katashi Okoshi, Marina P. Okoshi

**Affiliations:** ^1^ Botucatu Medical School Sao Paulo State University UNESP Botucatu Brazil; ^2^ School of Physical Therapy Federal University of Mato Grosso do Sul Campo Grande Brazil; ^3^ Institute of Biosciences Sao Paulo State University UNESP Botucatu Brazil

**Keywords:** echocardiogram, gastrocnemius, myocardial infarction, physical exercise

## Abstract

We compared the influence of aerobic and resistance exercise on cardiac remodelling, physical capacity and skeletal muscle oxidative stress in rats with MI‐induced heart failure. Three months after MI induction, Wistar rats were divided into four groups: Sham; sedentary MI (S‐MI); aerobic exercised MI (A‐MI); and resistance exercised MI (R‐MI). Exercised rats trained three times a week for 12 weeks on a treadmill or ladder. Statistical analysis was performed by ANOVA or Kruskal‐Wallis test. Functional aerobic capacity was greater in A‐MI and strength gain higher in R‐MI. Echocardiographic parameters did not differ between infarct groups. Reactive oxygen species production, evaluated by fluorescence, was higher in S‐MI than Sham, and lipid hydroperoxide concentration was lower in A‐MI than the other groups. Glutathione peroxidase activity was higher in A‐MI than S‐MI and R‐MI. Superoxide dismutase was lower in S‐MI than Sham and R‐MI. Gastrocnemius cross‐sectional area, satellite cell activation and expression of the ubiquitin‐proteasome system proteins did not differ between groups. In conclusion, aerobic exercise and resistance exercise improve functional capacity and maximum load carrying, respectively, without changing cardiac remodelling in infarcted rats. In the gastrocnemius, infarction increases oxidative stress and changes antioxidant enzyme activities. Aerobic exercise reduces oxidative stress and attenuates superoxide dismutase and glutathione peroxidase changes.

## 1 INTRODUCTION

Myocardial infarction (MI) is a leading cause of cardiac remodelling and heart failure, which are responsible for considerable mortality and morbidity worldwide.^1^ Heart failure affects several organs and tissues including skeletal muscles.^2^, ^3^ Clinical and experimental studies have shown that heart failure–induced skeletal myopathy plays an important role in symptoms such as early fatigue and exercise intolerance. Although several skeletal muscle abnormalities have been well characterized in heart failure,^4^, ^5^, ^6^ the mechanisms involved in muscle changes are not completely understood.^7^

Oxidative stress, which is increased in the myocardium and skeletal muscles during heart failure,^8^, ^9^ has been investigated as a causal factor in muscle changes.^10^, ^11^ Increased production of reactive oxygen species (ROS) in skeletal muscles of heart failure animals causes protein damage leading to hyperactivity of ubiquitin‐proteasome system (UPS) and is associated with muscle atrophy.^12^, ^13^ Despite remarkable progress in the therapeutic approach of heart failure, treatment of this syndrome remains a major challenge due to the high mortality rates**.** 1 Additionally, there is no specific therapy to prevent or recover from heart failure–associated skeletal muscle changes.

Current guidelines recommend regular physical exercise for patients with stable heart failure to prevent and/or attenuate cardiac remodelling and skeletal muscle alterations.^14^, ^15^ Several positive effects on cardiovascular function, functional capacity, inflammatory markers, antioxidant status and skeletal muscles have been described after aerobic exercise.^16^, ^17^, ^18^, ^19^, ^20^ Knowledge on the benefits of exercise in heart failure has primarily resulted from investigations with aerobic exercise as resistance training was not considered safe in heart failure.^16^ More recently, beneficial effects from resistance training have been described; these include increased maximal oxygen consumption and improved physical fitness and muscle strength and endurance.^16^, ^21^, ^22^ In addition, studies have provided evidence that resistance training can modulate human muscle mass by stimulating satellite cells to re‐enter the cell cycle and proliferate, resulting in muscle hypertrophy.^23^ However, studies on the effects of resistance exercise after MI are scarce.^22^, ^24^

Exercise is a strong modulator of oxidative stress. A single bout of intense exercise increases the formation of reactive oxygen species (ROS) leading to oxidative damage of cell components.^25^ On the other hand, regular exercise elevates ROS production to a level that may induce tolerable damage, which, in turn, up‐regulates cellular antioxidant systems stimulating oxidative damage repair systems.^26^ In this study, we compared the influence of aerobic and resistance exercise on cardiac remodelling, physical capacity and skeletal muscle oxidative stress in rats with myocardial infarction–induced heart failure.

## 2 MATERIALS AND METHODS

### 2.1 Experimental groups

Male Wistar rats weighing 200‐250 g were purchased from the Central Animal House of Botucatu Medical School, UNESP, and housed in a room under controlled temperature and light‐dark cycles. Food and water were supplied ad libitum. All experiments were approved by the Animal Experimentation Ethics Committee of Botucatu Medical School, UNESP, SP, Brazil, which follows the guidelines established by the Brazilian College for Animal Experimentation.

Myocardial infarction (MI) was induced by ligating the left anterior descending coronary artery.^27^ Three months later, rats underwent echocardiographic examination and exercise testing, as described below, and were then assigned to four groups: Sham‐operated (n = 20), sedentary MI (S‐MI, n = 26), aerobic exercise MI (A‐MI, n = 26) and resistance exercise MI (R‐MI, n = 21). After three months, rats were subjected to transthoracic echocardiogram and exercise testing, and killed the next day.

In previous studies,^2^, ^28^ we found that rats develop heart failure sixth months after myocardial infarction. Therefore, the physical training protocol was started 3 months after infarction induction and maintained for a period of 3 months.

### 2.2 Treadmill exercise testing

Exercise tolerance was evaluated before, 45 days after initiating exercise and at the end of the experiment. Each rat was tested individually. After 5‐min warm‐up at 5 m/min on treadmill, the animals were subjected to exercise at 8 m/min followed by increments of 3 m/min every 3 min until exhaustion, which was determined when the animal refused to run even after electric stimulation or was unable to co‐ordinate steps. Maximum running speed was recorded and total distance calculated.^19^, ^20^ Rats underwent 5 min/d test environment adaptation for 1 week before evaluation. Results from exercise testing at day 45 of training were used to adjust exercise intensity.

### 2.3 Aerobic exercise training

Group A‐MI was subjected to moderate‐intensity treadmill running 3 d/wk for 3 months. There was an adaptation period, with a gradual increase in speed and exercise duration. Speed from the 1st to the 5th week was 5, 7.5, 10, 12 and 15 m/min. Exercise duration from the 1st to the 5th week was 10, 15, 25, 30 and 40 min. From the 6th week on, each session consisted of 40 min of running at 60% maximum attained treadmill exercise test velocity.^12^ After 45 days of aerobic exercise training, animals were re‐evaluated for running performance to adjust exercise intensity.

### 2.4 Maximum carrying load

Maximum carrying load was assessed on a vertical ladder (1.00 m; 0.20 m, 0.5‐cm grid, 80° incline). During an adaptive period, the rats performed three climbing attempts, for three consecutive days, starting at different ladder locations: near the top, at the middle and at the lower base of the apparatus.^29^ Maximum carrying load was then evaluated for each rat by performing a maximum of 9 ladder climbs with progressively heavier loads. At the first attempt, rats climbed the ladder carrying a load equivalent to 75% of their bodyweight. After completing each climb, the load was increased by 15% bodyweight until a load was reached where the rats could not climb the entire ladder. The heaviest load that the rat successfully carried the entire length of the ladder was considered the maximum carrying load. Failure was determined when the rat could not progress up the ladder after three successive stimuli to the tail.^30^ Maximum carrying load test was performed before starting the resistance training protocol, 45 days after training for load adjustment (data not shown) and at the end of the experiment.

### 2.5 Resistance exercise training

The rats were subjected to a low‐volume resistance training protocol 3 days a week for 3 months. In the first week, rats performed 3 climbs with gradually increasing loads: no load on the first day, 15% rat bodyweight on the second day and 30% rat bodyweight on the third day. From the second week on, the protocol consisted of 4 climbs. The length of the ladder required the animals to make 8‐12 repetitions per climb. The climbs consisted of carrying progressive load of 50%, 75%, 90% and 100% of the maximal carrying load of each animal, with a 2‐minute rest between climbs at the housing chamber on the top of the ladder.^31^ After 45 days, rats were re‐evaluated to adjust training load.

### 2.6 Echocardiographic evaluation

After anaesthesia by intramuscular injection of a mixture of ketamine (50 mg/kg) and xylazine (1 mg/kg), echocardiogram was performed using an apparatus (Vivid S6, General Electric Medical Systems) equipped with a 5‐11.5 MHz multifrequency probe, as previously described.^32^, ^33^, ^34^

### 2.7 Infarct size

Left ventricle (LV) midventricular slices (5‐6 mm from the apex) were stained with picrosirius red. Infarction size was calculated by dividing the sum of endocardial and epicardial infarcted ventricular lengths by the sum of total (infarcted and viable myocardial) endocardial and epicardial ventricular circumferences as previously described.^28^ Rats with small MI size (<30%) were excluded from this study.

### 2.8 Collection of skeletal muscle and other tissues for analysis

Rats were anaesthetized with intraperitoneal sodium thiopental (50 mg/kg) and killed. After blood collection, hearts were removed by thoracotomy. Atria and ventricles were dissected and weighed. Gastrocnemius muscles from the right and left hind limbs were dissected, weighed, frozen in liquid nitrogen and stored at −80°C. Lung weight was used to assess the degree of pulmonary congestion. Fragments of lung and liver were weighed before and after drying sessions (65°C for 72 hours) to evaluate wet/dry weight ratio.

### 2.9 Skeletal muscle morphology

To evaluate skeletal muscle trophicity, 10‐µm‐thick serial transverse sections of the gastrocnemius mid‐belly were cut in a cryostat cooled to −20°C and stained with haematoxylin and eosin. At least 150 cross‐sectional fibre areas were measured from each muscle.^35^ Measurements were performed using a compound microscope (Leica DMLS) attached to a computerized imaging analysis system (Media Cybernetics).

### 2.10 Antioxidant enzyme activity and lipid hydroperoxide concentration

Gastrocnemius samples (∼100 mg) were homogenized in 2 mL of cold 0.1 mol/L phosphate buffer, pH 7.0, and centrifuged at 10 000 *g*, for 15 minutes at 4°C. The supernatant was assayed for total protein, lipid hydroperoxide and glutathione peroxidase (GSH‐Px, http://www.chem.qmul.ac.uk/iubmb/enzyme/EC1/11/1/9.html); catalase (http://www.chem.qmul.ac.uk/iubmb/enzyme/EC1/11/1/6/.html); and superoxide dismutase (SOD, http://www.chem.qmul.ac.uk/iubmb/enzyme/EC1/15/1/1/.html) activities by spectrophotometry.^19^

### 2.11 Reactive oxygen species generation

Oxidative fluorescent dye dihydroethidium (DHE) was used to evaluate in situ production of ROS. Ten‐µm‐thick gastrocnemius samples were cut in a cryostat. Sections were incubated in 10 μmol/L DHE (diluted in 0.01% methanol) for 30 minutes at 37°C in the dark and then washed three times in phosphate‐buffered saline (PBS), pH 7.4. To stain cell nuclei, sections were incubated with DAPI. The slides were again washed in PBS and coverslips allocated using ProLong Gold Antifade Reagent (Life Technologies). The slides were analysed under a specific microscope for immunofluorescence detection (Olympus BX51, equipped with Olympus U‐RFL‐T fluorescence emitter and Olympus DP72 camera, Panasonic).

### 2.12 Protein oxidation status

Oxidation status of gastrocnemius proteins was assessed by measuring carbonyl group levels using an OxyBlot Protein Oxidation Detection Kit (Millipore #S7150), following the manufacturer's protocol.

### 2.13 Protein expression

As an increase in muscle ROS production is associated with hyperactivation of the ubiquitin‐proteasome system^13^ resulting in increased proteolysis, we also analysed ubiquitin‐proteasome system protein expression by Western blotting.^36^, ^37^ We used specific anti‐20S proteasome (α5/α7, β1, β5 and β7 subunits, Abcam, Ref. ab22673), anti‐ubiquitin (Ub P4D1, Santa Cruz Biotechnology, Ref. sc‐8017) and anti‐Pax‐7 (Pax‐7 3/7 B‐5, Santa Cruz Biotechnology, Ref. sc‐365843) antibodies. Protein levels were normalized to GAPDH (6C5 sc‐32233, Santa Cruz Biotechnology). Gastrocnemius samples (~50 mg) were homogenized in 50 mmol/L Tris‐HCl, 1 mmol/L EDTA and protease inhibitor (Sigma Ref. S8820‐2TAB), pH 7.4, using zirconium beads (0.5 mm) for 5 minutes at 4ºC in a Bullet Blender^®^ homogenizer (Next Advance, Inc). The lysate was centrifuged at 12 000 *g* for 10 minutes at 4°C, and supernatant protein content was quantified by Bradford assay. Samples were separated on a polyacrylamide gel and transferred to a nitrocellulose membrane. After blockade (1 hour), membrane was incubated with the primary antibodies (overnight at 4ºC), washed with TBS and Tween‐20, and incubated with secondary peroxidase‐conjugated antibodies (90 minutes at room temperature). Immobilon^®^ Classico Western HRP Substrate (Merck Millipore, Ref. WBLUC0500) and image analyser ImageQuant LAS 4000 (GE Healthcare Life Sciences) were used to detect bound antibodies, which were quantified by densitometry using the program Gel Pro 3.1.

### 2.14 Satellite cell activation

To evaluate whether exercise induces muscle effects through satellite cell activation, we concluded our evaluation by analysing the expression of proteins that are satellite cell activation markers. Gastrocnemius samples were cut at 10 µm thickness in a cryostat cooled to −20°C. Sections were washed four times in PBS to remove Tissue‐Tek, fixed in methanol for 10 minutes at 4°C, washed three times and blocked in 5% BSA diluted in PBS for 30 minutes. The slides were then incubated with blocking solution containing the primary antibody (anti‐NCAM—sc10735, anti‐MyoD—sc760 and anti‐neonatal MHC—sc53097, Santa Cruz Biotechnology) overnight at 4°C. After washing with PBS, the slides were incubated with secondary antibody diluted in PBS for 1 hour at room temperature. The slides were washed and incubated with DAPI for 5 minutes. Coverslips were allocated using ProLong Gold Antifade Reagent (Life Technologies). The slides were analysed under a specific microscope for immunofluorescence detection.

### 2.15 Statistical analysis

Comparisons between groups were performed by one‐way analysis of variance complemented by Student‐Newman‐Keuls test for parametric variables, which are expressed as mean ± standard deviation. Non‐parametric variables were compared using the Kruskal‐Wallis test followed by Dunn's test and are expressed as median and percentiles. Data normality was evaluated by Shapiro‐Wilk test. Two independent variables were considered in this study: myocardial infarction in two levels, presence or absence; and exercise in three levels, sedentary, aerobic exercise and resistance exercise. Dependent variables consist of anatomical, echocardiographic, physical capacity, and skeletal muscle analysis data. The significance level was set at 5%.

## 3 RESULTS

### 3.1 Experimental groups

The following rats were excluded from this study due to LV infarcted area lower than 30% of total LV area in histological analysis: 13 in S‐MI, 7 in R‐MI and 12 in A‐MI. In addition, 7 rats died during the experimental period: 4 in S‐MI, 1 in R‐MI and 2 in A‐MI.

Anatomical data are shown in Figure S1. Bodyweight did not differ between groups. Atria and right ventricle (RV) weights, in absolute and normalized to bodyweight values, were higher in S‐MI and R‐MI than Sham. Right ventricle weight was lower in A‐MI than the other infarct groups. Gastrocnemius weight was lower in A‐MI than Sham, but did not differ between groups when normalized to bodyweight. We observed symptoms related to heart failure only in a few animals (Table S1).

**FIGURE 1:**
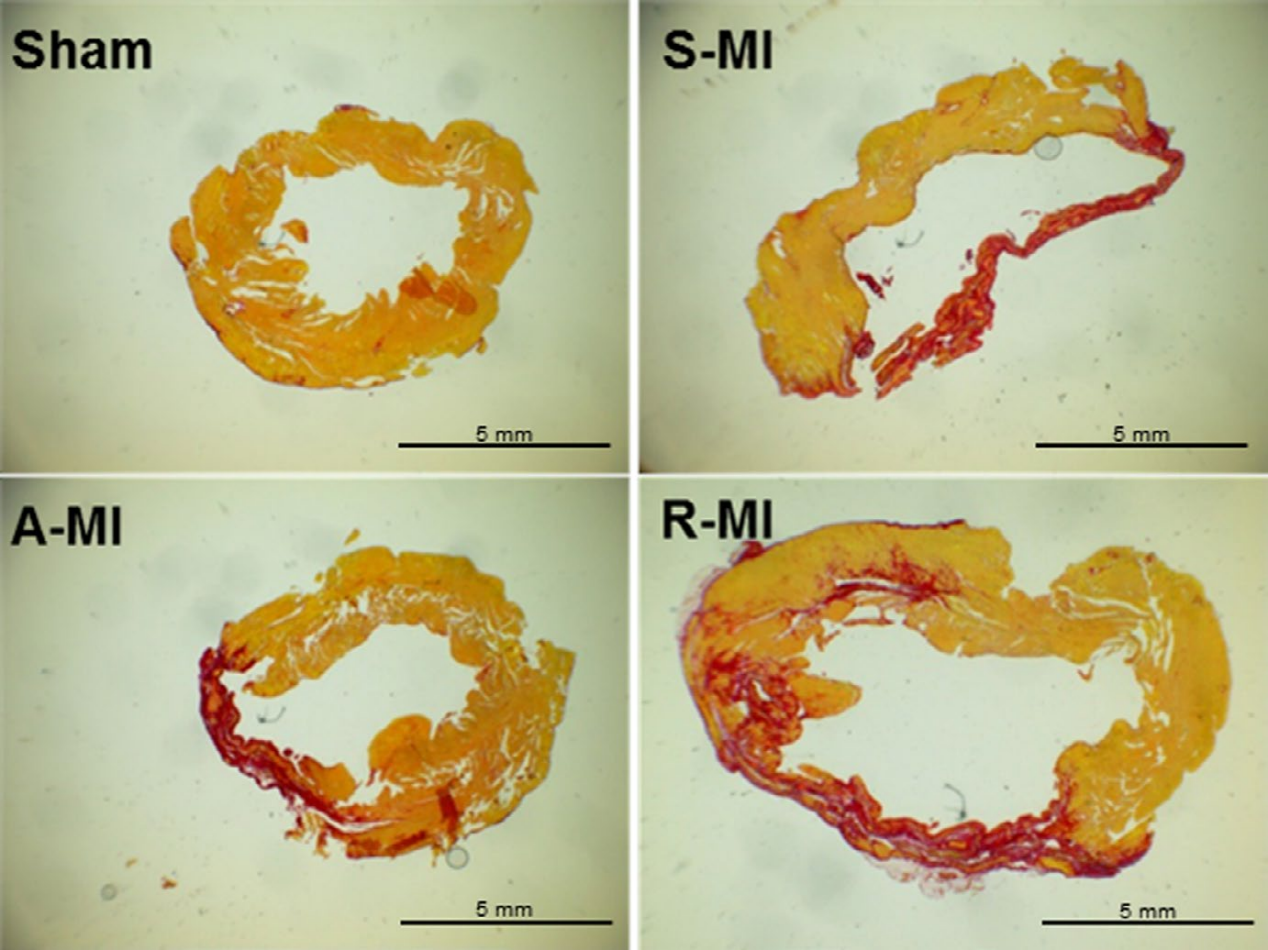
Representative histological sections of picrosirius red‐stained left ventricles from Sham, sedentary myocardial infarction (S‐MI), aerobic exercised MI (A‐MI) and resistance exercised MI (R‐MI) groups

Infarction size, measured by LV histological analysis, was similar between infarct groups (S‐MI 39.6 ± 5.9%; A‐MI 42.8 ± 8.9%; and R‐MI 38.2 ± 6.4%, *P* > .05; Figure 1).

### 3.2 Maximum exercise capacity and maximum carrying load test

**FIGURE 2:**
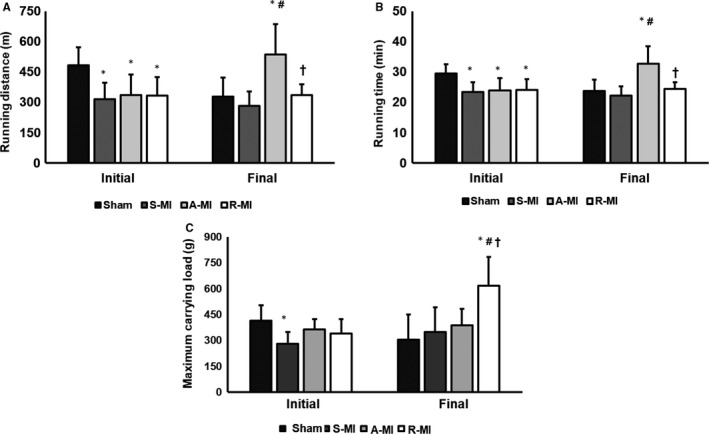
Initial and final maximum exercise test (A, B) and maximum carrying load test (C). A‐MI, aerobic exercised MI (n = 7); R‐MI, resistance exercised MI (n = 11); S‐MI, sedentary myocardial infarction (MI; n = 7); and Sham, n = 4. Data are mean ± SD; ANOVA and Student‐Newman‐Keuls; *P < .05 vs Sham; ^#^ *P* < .05 vs S‐MI; ^†^ *P* < .05 vs A‐MI

In the initial exercise capacity test, infarct groups had lower run distance and time on the treadmill than Sham. There were no differences between infarct groups. In the final test, functional capacity was better in A‐MI than the other groups (Figure 2A, B). In the initial test, S‐MI had lower load‐carrying capacity than Sham. At the end of the experiment, R‐MI carried up more load than the other groups (Figure 2C).

### 3.3 Echocardiographic evaluation

Before exercise, infarcted rats had LV hypertrophy characterized by higher systolic and diastolic diameters, wall thickness, mass, and systolic and diastolic areas than Shams, with systolic dysfunction characterized by reduced endocardial fractional shortening, posterior wall shortening velocity, fractional area change and increased myocardial performance index (Table S2). Final echocardiographic data are presented in Table 1. Infarct groups maintained LV hypertrophy with systolic dysfunction as observed before exercise. R‐MI had a lower LV relative wall thickness than A‐MI. Diastolic function did not differ between groups. No differences were observed between infarct groups.

**TABLE 1:** Final echocardiographic data

	Sham (n = 20)	S‐MI (n = 9)	A‐MI (n = 9)	R‐MI (n = 13)
BW (g)	548 (519‐574)	531 (506‐574)	507 (464‐556)	526 (476‐573)
LVDD (mm)	8.31 ± 0.41	11.1 ± 0.88*	10.3 ± 0.89*	10.9 ± 0.82*
LVDD/BW (mm/kg)	15.0 (14.4‐16.3)	21.5 (18.5‐22.2)*	20.4 (19.3‐20.8)*	20.8 (19.8‐22.4)*
LVSD (mm)	4.14 (3.96‐4.30)	8.92 (8.33‐9.65)*	7.56 (6.71‐8.93)*	8.71 (7.83‐9.36)*
DPWT (mm)	1.40 (1.38‐1.45)	1.80 (1.63‐2.03)*	1.86 (1.69‐2.00)*	1.69 (1.59‐1.73)*
DSWT (mm)	1.42 ( 1.40‐1.45)	1.69 (1.35‐1.89)	1.84 (1.33‐1.99)	1.57 (1.31‐1.78)
RWT	0.35 (0.33‐0.36)	0.32 (0.30‐0.36)	0.36 (0.34‐0.38)	0.31 (0.28‐0.32)*, †
AO (mm)	4.20 ± 0.16	4.01 ± 0.23	4.02 ± 0.13	4.06 ± 0.27
LA (mm)	5.66 (5.29‐6.13)	8.76 (6.82‐9.23)*	7.23 (7.04‐7.61)*	8.10 (6.85‐8.74)*
LA/AO	1.37 (1.25‐1.43)	2.22 (1.60‐2.36)*	1.81 (1.74‐1.84)*	1.86 (1.78‐2.24)*
LA/BW (mm/kg)	10.5 (9.22‐11.1)	15.9 (11.8‐17.6)*	14.1 (12.8‐15.7)*	14.6 (12.6‐17.2)*
LVM (g)	0.85 (0.76‐0.92)	1.66 (1.44‐2.07)*	1.39 (1.30‐1.98)*	1.61 (1.37‐1.79)*
LVMI (g/kg)	1.53 (1.46‐1.70)	3.32 (2.63‐4.11)*	3.03 (2.72‐3.99)*	3.07 (2.49‐3.49)*
End‐DA (mm^2^)	49.2 (47.2‐51.2)	89.9 (83.7‐98.7)*	79.3 (67.1‐105)*	93.5 (78.6‐107)*
End‐SA (mm^2^)	14.7 (14.1‐17.6)	64.5 (54.0‐67.5)*	49.5 (44.2‐71.3)*	59,5 (46.3‐77.1)*
MI size (%)	—	41.2 ± 7.75	36.5 ± 5.64	36.1 ± 9.44
HR (bpm)	276 ± 38.8	301 ± 32.3	292 ± 37.2	292 ± 20.5
EFS (%)	50.1 ± 3.58	19.8 ± 6.28*	26.3 ± 10.9*	21.1 ± 6.45*
PWSV (mm/s)	41.1 ± 5.68	25.4 ± 9.35*	29.9 ± 6.56*	28.0 ± 6.20*
Tei index	0.46 ± 0.06	0.62 ± 0.14*	0.61 ± 0.09*	0.66 ± 0.12*
FAC (%)	68.5 ± 4.73	29.7 ± 8.92*	33.6 ± 9.35*	34.5 ± 12.2*
TDI S (average, cm/s)	3.55 ± 0.36	2.80 ± 0.49*	3.11 ± 0.70*	2.93 ± 0.45*
Mitral E (cm/s)	77.0 (73.0‐85.0)	102 (75.8‐123)	80.0 (66.0‐85.0)	77.5 (71.0‐122)
Mitral A (cm/s)	50.1 ± 15.8	38.6 ± 27.7	59.4 ± 23.2	47.3 ± 20.8
E/A	1.71 (1.36‐1.82)	4.27 (1.27‐6.12)	1.16 (0.91‐1.54)	1.41 (1.23‐5.77)
IVRT (ms)	25.8 ± 3.19	26.6 ± 5.20	29.0 ± 6.91	27.5 ± 4.27
IVRTn	52.6 (49.7‐61.1)	57.0 (49.0‐69.0)	63.7 (54.7‐74.2)	59.3 (53.2‐68.5)
EDT (ms)	51.0 (48.0‐55.8)	33.0 (33.0‐51.0)	47.0 (30.8‐62.5)	39.5 (37.0‐53.0)
TDI E’ (average, cm/s)	4.16 ± 0.68	4.18 ± 0.76	3.74 ± 1.11	3.92 ± 0.56
E/TDI E’ (average)	18.4 (16.4‐21.7)	22.5 (20.4‐24.9)	21.6 (16.1‐23.9)	21.8 (18.8‐27.8)
E’/A’ (cm/s)	1.29 ± 0.54	1.38 ± 0.42	1.05 ± 0.49	1.07 ± 0.30

Note

Data are mean ± SD or median and percentiles; ANOVA and Student‐Newman‐Keuls or Kruskal‐Wallis test.

Abbreviations: A‐MI, aerobic exercised MI; AO, aorta diameter; BW, bodyweight; DPWT, LV diastolic posterior wall thickness; DSWT, LV diastolic septal wall thickness, respectively; E/A, ratio between early (E)‐to‐late (A) diastolic mitral inflow; EDT, E‐wave deceleration time; EFS, endocardial fractional shortening; End‐DA, LV end‐diastolic area; End‐SA, LV end‐systolic area; FAC, fractional area change; HR, heart rate; IVRT, isovolumetric relaxation time; IVRTn, IVRT normalized to heart rate; LA, left atrial diameter; LVDD and LVSD, left ventricular (LV) diastolic and systolic diameters, respectively; LVM, LV mass; LVMI, LVM index; MI, myocardial infarction; N, number of animals; PWSV, posterior wall shortening velocity; R‐MI, resistance exercised MI; RWT, relative wall thickness; S‐MI, sedentary myocardial infarction (MI); TDI E’ and A’, TDI of early (E’) and late (A’) diastolic velocity of mitral annulus; TDI S, tissue Doppler imaging (TDI) of systolic velocity of the mitral annulus; Tei index, myocardial performance index.

* *P* < .05 vs Sham;

^†^ *P* < .05 vs A‐MI.

### 3.4 Morphometric study

**FIGURE 3:**
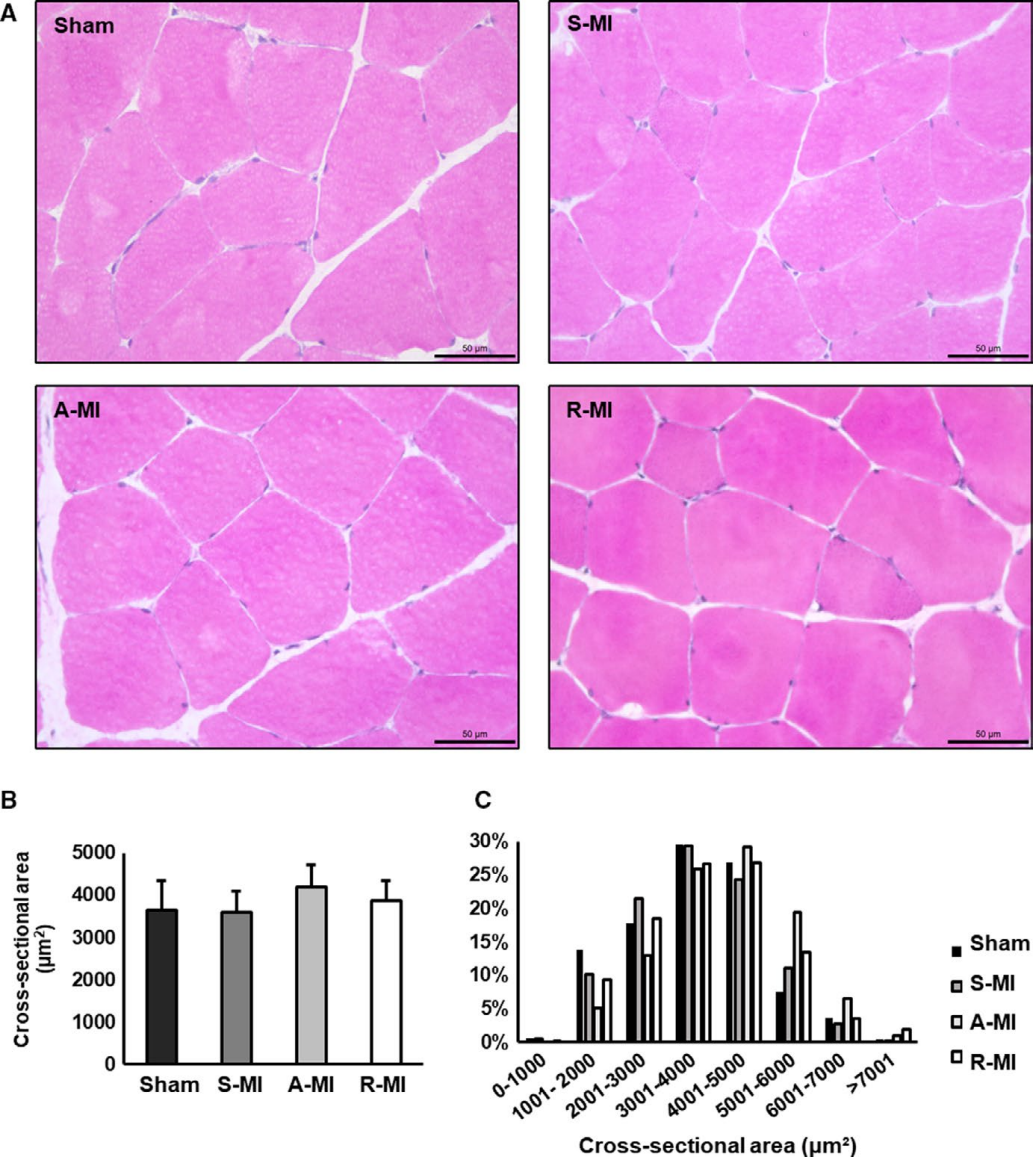
Haematoxylin‐ and eosin‐stained gastrocnemius sections (A). Objective: 40X. Gastrocnemius cross‐sectional areas (B). Gastrocnemius fibre cross‐sectional area distribution (C). A‐MI, aerobic exercised MI (n = 5); R‐MI, resistance exercised MI (n = 7); S‐MI, sedentary myocardial infarction (MI; n = 8); and Sham, n = 6. Data are mean ± SD; ANOVA; *P* > .05

Gastrocnemius fibre cross‐sectional area did not differ between groups (Sham 3,660 ± 701, S‐MI 3,594 ± 500, A‐MI 4,200 ± 521 and R‐MI 3,885 ± 462 µm^2^; *P* > .05). Representative histological sections are shown in Figure 3.

### 3.5 Reactive oxygen species generation

**FIGURE 4:**
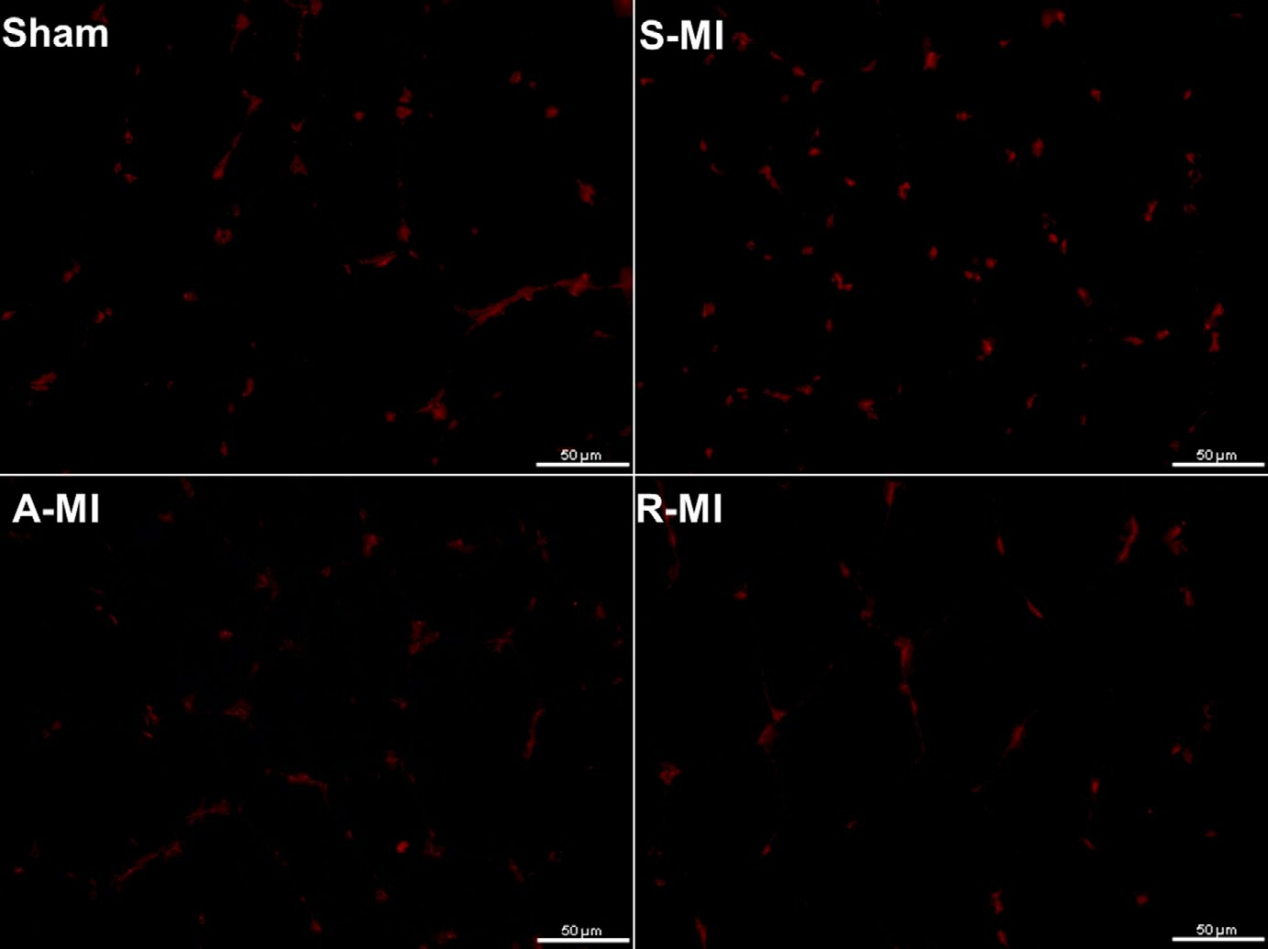
Gastrocnemius sections stained by immunofluorescence for ethidium (red), which is the product resulting from oxidation of dihydroethidium by superoxide. Objective 40x. A‐MI, aerobic exercised MI (n = 5); R‐MI, resistance exercised MI (n = 7); S‐MI, sedentary myocardial infarction (MI, n = 8); Sham, n = 6. ANOVA and Student‐Newman‐Keuls, *P* < .05 S‐MI vs Sham

Muscular in situ generation of ROS was higher in S‐MI than Sham (Sham 1.00 ± 0.78; S‐MI 1.97 ± 0.39; A‐MI 1.35 ± 0.63; and R‐MI 1.50 ± 0.47 arbitrary units; Figure 4).

### 3.6 Lipid hydroperoxide concentration and antioxidant enzyme activity

**FIGURE 5:**
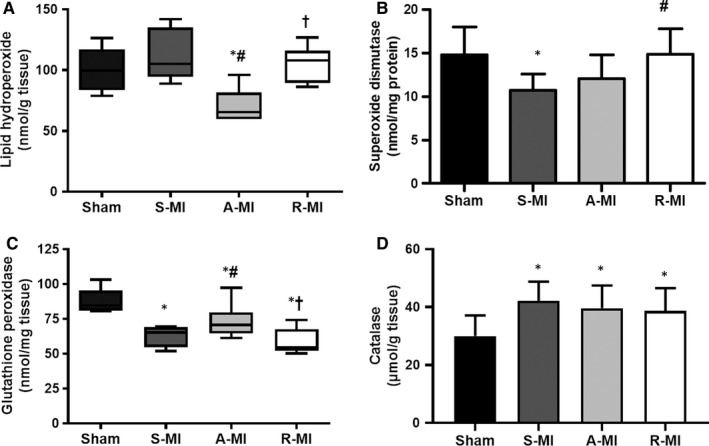
Gastrocnemius lipid hydroperoxide concentration (A) and antioxidant enzyme activities (B‐D). A‐MI, aerobic exercised MI; R‐MI, resistance exercised MI; n = 8 animals per group; S‐MI, sedentary myocardial infarction (MI). Data are mean ± SD or median and percentiles; ANOVA and Student‐Newman‐Keuls or Kruskal‐Wallis test; *P < .05 vs Sham; ^#^ *P* < .05 vs S‐MI; ^†^ *P* < .05 vs A‐MI

Gastrocnemius lipid hydroperoxide concentration was lower in A‐MI than the other groups (Figure 5A). Superoxide dismutase activity was lower in S‐MI than Sham and R‐MI groups. Glutathione peroxidase activity was lower in infarct groups than Sham and higher in A‐MI than S‐MI and R‐MI. Catalase activity was increased in infarct groups (Figure 5B‐D).

### 3.7 Protein oxidation

Carbonyl group levels, measured in 25 to 100 kD molecular weight, did not differ between groups (Table S3).

### 3.8 Western blotting

Protein expression of 20S proteasome, ubiquitin and Pax‐7 did not differ between groups (Tables S3 and S4).

### 3.9 Satellite cell activation

**FIGURE 6:**
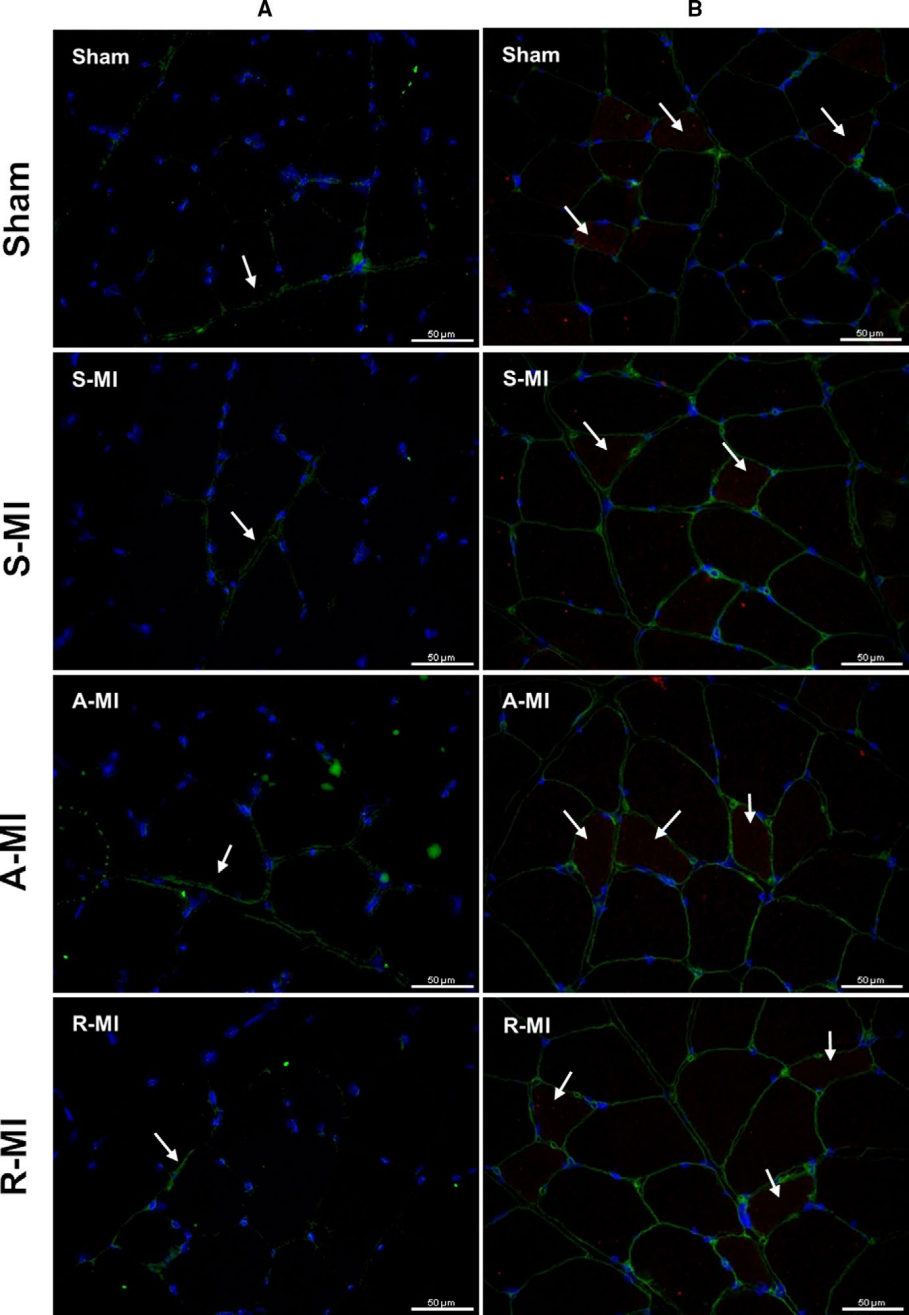
Gastrocnemius sections stained by immunofluorescence for NCAM (green) and DAPI for nuclei stain (blue) (A) or neonatal myosin heavy chain (MHC, red), wheat germ agglutinin (WGA) for cell membrane stain (green), and DAPI (B). Arrows indicate the presence of target proteins. Objective 40X. A‐MI, aerobic exercised MI (n = 5); R‐MI, resistance exercised MI (n = 7); S‐MI, sedentary myocardial infarction (MI, n = 8); Sham, n = 6

Satellite cell activation, evaluated by immunofluorescence staining for NCAM [Sham 0.86 (0.86‐1.07), S‐MI 0.43 (0.00‐0.86), A‐MI 0.86 (0.43‐2.14) and R‐MI 1.29 (0.86‐1.93)], neonatal myosin heavy chain [Sham 0.75 (0.75‐1.50), S‐MI 0.75 (0.19‐1.50), A‐MI 0.75 (0.38‐1.50) and R‐MI 0.75 (0.75‐1.50)] and MyoD [Sham 0.67 (0.67‐1.50), S‐MI 0.33 (0.00‐0.67), A‐MI 0.67 (0.00‐1.00) and R‐MI 0.67 (0.00‐1.00)] did not differ between groups (Figure 6).

## 4 DISCUSSION

In this study, we showed for the first time that moderate‐intensity aerobic exercise is more effective than resistance exercise in preventing an increase in reactive oxygen species and attenuating antioxidant system changes in skeletal muscle of infarcted rats.

Experimental myocardial infarction in rats is often used to induce cardiac remodelling and heart failure. This model has the advantage of slow cardiac remodelling development, which is usually observed in clinical settings.^28^ However, only rats with moderate to large infarction sizes develop LV dysfunction and heart failure.^28^ Therefore, to study rats with infarction sizes greater than 30% of the total LV area, we had to exclude a great number of infarcted rats.

The aerobic exercise protocol we applied is considered of moderate intensity. Our results showing that moderate‐intensity exercise improved functional capacity compared to sedentary infarcted and Sham rats confirm data from experimental and clinical studies.^12^, ^38^ Similarly, the resistance exercise training was effective in improving maximum load‐carrying capacity above sedentary infarcted and Sham groups. As expected, aerobic training did not improve load‐carrying capacity and resistance training did not increase functional capacity.

Cardiac remodelling was evaluated by transthoracic echocardiogram. The infarct groups had similar infarct sizes and LV impairment before exercise (data not shown). At the end of the study, the infarct groups maintained the same cardiac remodelling pattern, characterized by LV hypertrophy and dilation with systolic dysfunction, observed before exercise. Systolic dysfunction was severe in infarct groups; LV fractional area change, an important index to evaluate LV systolic function in rats after infarction,^27^ was approximately 50% lower in infarcted rats than Sham. LV structural and functional changes were not attenuated by either aerobic or resistance exercise protocols.

These results are divergent from experimental studies showing that aerobic exercise improved LV dysfunction in different heart injury models.^12^, ^24^, ^39^, ^40^, ^41^ As exercise in this study was initiated three months after infarction, it is probable that the severe degrees of LV dilation and systolic dysfunction prevented a reverse remodelling process. The effects of resistance training on cardiac remodelling have undergone less study than aerobic exercise. As resistance exercise elevates arterial pressure, physicians are concerned that the increased afterload could impair LV dilation and cardiac remodelling.^42^ Our results allow us to conclude that resistance exercise is safe in post‐infarction rats. Similar to our results, Grans et al^22^ did not observe significant changes in LV function three months after resistance exercise in post‐infarction rats. On the other hand, Cai et al^24^ showed that resistance exercise improved cardiac function. The divergence between studies on the effects of exercise on ventricular function is probably related to differences in infarction size and time after infarction when physical training is initiated.

As cardiac remodelling did not differ between infarct groups, the better functional and load‐carrying capacity, in A‐MI and R‐MI groups, respectively, were probably related to improvements in skeletal muscles properties.

Oxidative stress occurs when there is an imbalance between free radical generation and antioxidant systems. In this study, we extensively evaluated oxidative stress in the gastrocnemius muscle, a glycolytic muscle. The gastrocnemius is a glycolytic muscle, whose antioxidant defences are lower than oxidative muscles, thus making it more susceptible to ROS damage.^13^

Lipid hydroperoxide concentration is an oxidative stress biomarker; it was lower in A‐MI than the other groups, showing that only aerobic exercise had a protective effect against skeletal muscle oxidative stress. However, when evaluating tissue $O_{2}^{-}$ levels by dihydroethidium fluorescence, increased $O_{2}^{-}$ levels were observed in S‐MI than Sham, suggesting that both exercise protocols prevented changes in ROS production. We have previously shown that aerobic exercise prevented increased oxidative stress in soleus muscle and the myocardium of aortic stenosis rats.^19^, ^20^

Superoxide dismutase represents the first defence against superoxide radical anion catalysing the dismutation of superoxide in hydrogen peroxide in the presence of the H^+^ ion.^43^ Superoxide dismutase activity was lower in S‐MI than Sham and R‐MI. A similar result was observed ^24^ in infarcted rats. Catalase activity was increased in infarct groups. Glutathione peroxidase activity was lower in infarct groups, and this decrease was attenuated by aerobic exercise. Considering that both catalase and glutathione peroxidase catalyse the formation of H_2_O and oxygen from H_2_O_2_ formed by the reaction of superoxide dismutase,^43^ increased catalase is probably a compensatory mechanism against the decreased glutathione peroxidase in the infarcted rats. Previous clinical and experimental studies have shown reduced antioxidant enzyme expression and activity in skeletal muscles during heart failure.^8^, ^44^

As increased muscle ROS production was associated with ubiquitin‐proteasome system hyperactivation in heart failure,^13^, ^45^ we also analysed oxidant status of proteins and protein expression of the ubiquitin‐proteasome system, which did not differ between groups. The divergent results are probably related to evaluated animal—rat versus mouse. Finally, considering the adaptive potential of skeletal muscle due to its high plasticity and regenerative capacity under exercise,^46^ we evaluated the expression of proteins that are markers of satellite cell activation. We observed that myocardial infarction or exercise had no influence in those markers of satellite cell activation. As activation of the ubiquitin‐proteasome system and satellite cell is related to muscle atrophy, our negative results are in accordance with the absence of gastrocnemius atrophy. Skeletal muscle atrophy is usually observed in severe heart failure.^47^

In conclusion, aerobic and resistance exercise improves functional capacity and maximum load carrying, respectively, without changing cardiac remodelling in infarcted rats. In the gastrocnemius muscle, infarction increases ROS and changes antioxidant enzyme activities. Resistance exercise attenuates ROS production and preserves superoxide dismutase activity. Aerobic exercise reduces muscle lipid hydroperoxide concentration and attenuates ROS production and superoxide dismutase and glutathione peroxidase changes. These results allow us to raise the hypothesis that aerobic exercise is superior to resistance exercise against oxidative stress.

## CONFLICT OF INTEREST

The authors declare that they have no competing interests.

## AUTHORS CONTRIBUTION

MJG and MPO contributed to study design, manuscript writing and fundraising; LUP, ARRL, DRAR, PFM, FCD, THDP, EAR, LMS, IFT, AAHF, LAMZ and KO contributed to data collection. All authors have approved the final manuscript.

## CONSENT TO PUBLISH

All authors gave consent for manuscript publication.

## Supporting information

Figure S1

Table S1

Table S2

Table S3

Table S4

## Data Availability

The data sets used and/or analysed during the current study are available from the corresponding author on request.
