# Supplementary figures and images for: Effects of aerobic and resistance exercise on cardiac remodelling and skeletal muscle oxidative stress of infarcted rats
Source: J Cell Mol Med. 2020 Apr 2;24(9):5352–62. doi: 10.1111/jcmm.15191 (PMC7205792; doi:10.1111/jcmm.15191)

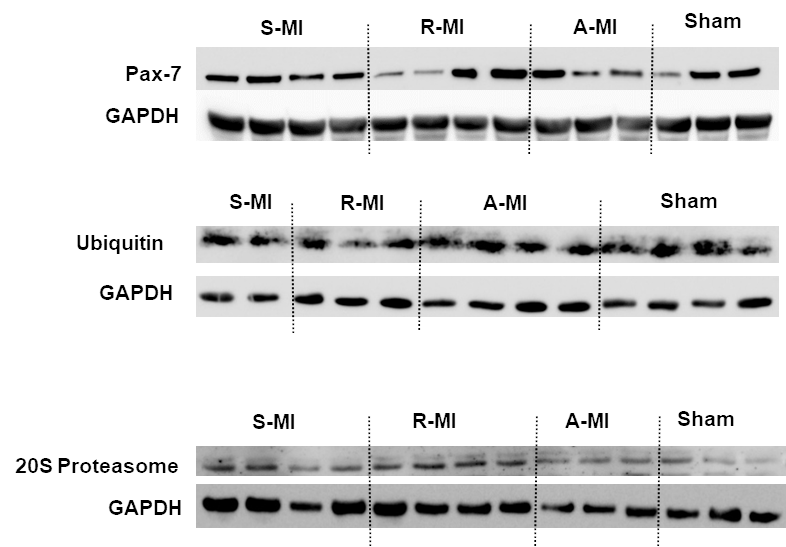

Supplement: Supplementary file 1 — Figure S1 [file JCMM-24-5352-s001.tiff]
