# Supplementary material for: Effects of aerobic and resistance exercise on cardiac remodelling and skeletal muscle oxidative stress of infarcted rats
Source: J Cell Mol Med. 2020 Apr 2;24(9):5352–62. doi: 10.1111/jcmm.15191 (PMC7205792; doi:10.1111/jcmm.15191)
Supplement: Supplementary file 2 — Table S1 [file JCMM-24-5352-s002.docx]

**Supporting information 1.** Anatomical data

|  | **Sham**  **(n=20)** | **S-MI**  **(n=09)** | **A-MI**  **(n=09)** | **R-MI**  **(n=13)** |
| --- | --- | --- | --- | --- |
| **BW (g)** | 532 ± 47 | 544 ± 63 | 511 ± 53 | 528 ± 78 |
| **LV (W/D)** | 4.05 (3.78-4.26) | 4.38 (3.94-5.10) | 4.00 (3.77-4.42) | 4.20 (4.08-4.28) |
| **RV weight (g)** | 0.23 (0.21-0.26) | 0.36 (0.29-0.47)* | 0.22 (0.20-0.27)^#^ | 0.34 (0.30-0.52)*† |
| **RV/BW (mg/g)** | 0.44 (0.0-0.47) | 0.71 (0,52-0.90)* | 0.46 (0.36-0.56) | 0.65 (0.55-1.01)*† |
| **RV (U/S)** | 4.05 (3.80-4.26) | 4.38 (3.94-5.10) | 4.00 (3.77-4.42) | 4.20 (4.08-4.28) |
| **Atria** | 0.10 (0.08-0.11) | 0.15 (0.11-0.31)* | 0.15 (0.10-0.21) | 0.17 (0.14-0.23)* |
| **Atria/BW (mg/g)** | 0.20 (0.15-0.24) | 0.32 (0.21-0.54)* | 0.27 (0.20-0.35) | 0.31 (0.23-0.46)* |
| **Atria (W/D)** | 4.18 ± 0.85 | 4.50 ± 1.25 | 4.48 ± 0.95 | 4.23 ± 0.70 |
| **Lung (g)** | 1.93 ± 0.62 | 2.98 ± 0.89* | 2.39 ± 0.53 | 2.64 ± 0.70* |
| **Lung/BW (mg/g)** | 3.61 ± 1.05 | 5.62 ± 1.92* | 4.67 ± 1.00 | 5.20 ± 1.97* |
| **Lung (W/D)** | 4.49 (4.38-4.54) | 4.28 (3.89-4.87) | 4.87 (4.30-5.32) | 4.50 (4.17-4.90) |
| **Gastrocnemius (g)** | 2.85 (2.57-3.03) | 2.50 (2.38-2.85) | 2.40 (2.13-2.60)* | 2.67 (2.29-2.91) |
| **Gastrocnemius/BW (mg/g)** | 5.45 (4.70-5.86) | 4.86 (4.45-5.09) | 4.50 (4.42-5.40) | 4.84 (4.54-5.19) |

S-MI: sedentary myocardial infarction (MI); A-MI: aerobic exercised MI; R-MI: resistance exercised MI; n: number of animals; BW: body weight; LV: left ventricle; RV: right ventricle; W/D: wet/dry weight ratio. Data are mean ± SD or median and percentiles; ANOVA and Student-Newman-Keuls or Kruskal-Wallis; *p<0.05 vs Sham; ^#^p<0.05 vs S-MI; ^†^p<0.05 vs A-MI.
