# Supplementary material for: Effects of aerobic and resistance exercise on cardiac remodelling and skeletal muscle oxidative stress of infarcted rats
Source: J Cell Mol Med. 2020 Apr 2;24(9):5352–62. doi: 10.1111/jcmm.15191 (PMC7205792; doi:10.1111/jcmm.15191)
Supplement: Supplementary file 3 — Table S2 [file JCMM-24-5352-s003.docx]

**Supporting information 2.** Frequency of heart failure features in the infarcted rats

|  | **S-MI**  **(n=9)** | **A-MI**  **(n=9)** | **R-MI**  **(n=13)** |
| --- | --- | --- | --- |
| **Atrial thrombi** | 11% (1) | 22% (2) | 23% (3) |
| **Pleuropericardial effusion** | 11% (1) | 22% (2) | 30% (4) |
| **Pulmonary congestion** | 44% (4) | 11% (1) | 23% (3) |
| **Right ventricular hypertrophy** | 33% (3) | ⎯ | 30% (4) |
| **Ascites** | 22% (2) | 44% (4) | 15% (2) |

S-MI: sedentary myocardial infarction (MI); A-MI: aerobic exercised MI; R-MI: resistance exercised MI; n: number of animals. Goodman test. Significance level was set at 5%.
