# Supplementary material for: Effects of aerobic and resistance exercise on cardiac remodelling and skeletal muscle oxidative stress of infarcted rats
Source: J Cell Mol Med. 2020 Apr 2;24(9):5352–62. doi: 10.1111/jcmm.15191 (PMC7205792; doi:10.1111/jcmm.15191)
Supplement: Supplementary file 4 — Table S3 [file JCMM-24-5352-s004.docx]

**Supporting information 3.** Initial echocardiographic data

|  | **Sham**  **(n=20)** | **S-MI**  **(n=09)** | **A-MI**  **(n=09)** | **R-MI**  **(n=13)** |
| --- | --- | --- | --- | --- |
| **BW (g)** | 463 ± 41.9 | 458 ± 42.7 | 443 ± 42.4 | 447 ± 46.9 |
| **LVDD (mm)** | 8.02 (7.74-8.37) | 10.4 (10.0-10 .9)* | 9.64 (9.05-10.2)* | 9.79 (9.63-10.4)* |
| **LVDD/BW (mm/Kg)** | 17.7 (16.9-18.5) | 23.2 (21.1-25.2)* | 23.5 (19.2-24.7)* | 21.9 (20.9-26.2)* |
| **LVSD (mm)** | 4.10 (3.85-4.32) | 8.16 (7.97-8.69)* | 7.16 (6.50-8.55)* | 7.89 (7.15-8.62)* |
| **DPWT (mm)** | 1.32 (1.27-1.37) | 1.65 (1.52-1.96)* | 1.57 (1.48-1.73)* | 1.65 (1.49-1.76)* |
| **DSWT (mm)** | 1.33 (1.27-1.37) | 1.53 (1.30-1.98) | 1.35 (1.19-1.44) | 1.40 (1.27-1.63) |
| **RWT** | 0.33 (0.32-0.34) | 0.31 (0.30-0.35) | 0.34 (0.29-0.38) | 0.32 (0.30-0.37) |
| **AO (mm)** | 4.01 (3.83-4.01) | 3.94 (3.74-4.01) | 3.83 (3.81-3.97) | 3,83 (3,57-4,01) |
| **LA (mm)** | 5.38 (5.15-5.66) | 7.66 (6.66-8.50)* | 6.57 (6.09-6.86)* | 6.75 (6.18-7.56)* |
| **LA/AO** | 1.36 (1.30-1.41) | 1.94 (1.66-2.33)* | 1.67 (1.53-1.78)* | 1.80 (1.53-2.04)* |
| **LA/BW (mm/Kg)** | 11.8 (10.5-12.7) | 17.4 (13.9-19.6)* | 14.1 (13.2-16.4)* | 15.0 (12.9-17.2)* |
| **LVM (g)** | 0.73 (0.66-0.80) | 1.53 (1.21-1.75)* | 1.15 (1.07-1.24)* | 1.13 (1.08-1.73)* |
| **LVMI (g/Kg)** | 1.56 (1.51-1.68) | 3.58 (2.63-3.97)* | 2.64 (2.43-2.96)* | 2.74 (2.42-3.73)* |
| **End-DA(mm^2^)** | 45.5 (37.1-49.4) | 86.1 (77.8-101)* | 71.5 (67.5-86.0)* | 77.60 (71.9-90.6)* |
| **End-SA. (mm^2^)** | 15.8 (12.5-17.7) | 61.6 (55.2-74.5)* | 50.4 (37.6-65.5)* | 52.2 (42.8-60.4)* |
| **MI size (%)** | Sham | 41.2 ± 7.61 | 37.9 ± 10.8 | 37.6 ± 6.72 |
| **HR (bpm)** | 284 ± 25.6 | 295 ± 26.1 | 290 ± 54.0 | 275 ± 23.9 |
| **EFS (%)** | 48.8 ± 3.77 | 20.7 ± 6.20* | 23.2 ± 5.81* | 21.9 ± 6.24* |
| **PWSV (mm/s)** | 39.8 ± 5.44 | 24.5 ± 7.67* | 31.2 ± 8.53* | 28.6 ± 8.37* |
| **Tei index** | 0.45 (0.43-0.53) | 0.74 (0.61-0.89)* | 0.70 (0.54-0.80)* | 0.76 (0.62-0.82)* |
| **FAC (%)** | 66.2 (62.7-69.2) | 26.1 (20.3-37.4)* | 28.2 (26.5-43.6)* | 33.1 (29.1-40.1)* |
| **TDI S (average cm/s)** | 3.47 ± 0.33 | 3.13 ± 0.36 | 3.18 ± 0.55 | 2.90 ± 0.46* |
| **Mitral E (cm/s)** | 78.5 (75.0-84.8) | 99.0 (89.0-108)* | 77.5 (68.8-90.5)^#^ | 83.0 (77.0-100) |
| **Mitral A (cm/s)** | 47.0 (44.0-53.0) | 44.0 (21.0-65.0) | 54.5 (41.0-81.8) | 42.0 (22.5-58.0) |
| **E/A** | 1.70 (1.49-1.87) | 2.02 (1.38-5.55) | 1.34 (1.15-1.76) | 1.58 (1.39-3.86) |
| **IVRT (ms)** | 26.0 (22.0-26.0) | 30.0 (25.0-33.0) | 33.0 (30.0-37.0)* | 30.0 (24.0-32.5) |
| **IVRTn** | 54.6 ± 6.72 | 63.0 ± 10.7* | 70.7 ± 11.1* | 62.0 ± 13.3 |
| **EDT (ms)** | 48.5 (45.0-56.0) | 39.0 (35.5-46.3) | 41.0 (36.0-55.0) | 43.0 (37.0-58.3) |
| **TDI E’ (average cm/s)** | 4.15 ± 0.72 | 4.10 ± 0.52 | 3.66 ± 0.47 | 3.76 ± 0.50 |
| **TDI A’ (average cm/s)** | 3.18 ± 0.85 | 3.33 ± 1.03 | 3.73 ± 1.23 | 3.02 ± 0.71 |
| **E/TDI E’ (average)** | 19.8 ± 3.69 | 24.5 ± 5.38* | 21.3 ± 3.22 | 24.5 ± 6.44* |

S-MI: sedentary myocardial infarction group; A-MI: aerobic exercise myocardial infarction group; R-MI: resistance exercise myocardial infarction group; n: number of animals. BW: body weight; LVDD and LVSD: left ventricular (LV) diastolic and systolic diameter, respectively. DPWT: LV diastolic posterior wall thickness; DSWT: LV diastolic septal wall thickness, respectively; RWT: relative wall thickness; AO: aorta diameter; LA: left atrial diameter; LVM: LV mass; LVMI: LVM index; MI: myocardial infarction; HR: heart rate; EFS: endocardial fractional shortening; MFS: midwall fractional shortening; PWSV: posterior wall shortening velocity; Tei index: myocardial performance index; EF: ejection fraction; FCA: fractional area change; TDI S: tissue Doppler imaging of systolic velocity of the mitral annulus; E/A: ratio between early- (E-) to-late (A) diastolic mitral inflow; IVRT: isovolumetric relaxation time; IVRTn: IVRT normalized to heart rate; EDT: E wave deceleration time; TDI E’ and A’: tissue Doppler imaging (TDI) of early (E’) and late (A’) diastolic velocity of mitral annulus. Data are mean ± SD or median and percentiles; ANOVA and Student-Newman-Keuls or Kruskal-Wallis. * p<0.05 vs Sham.
