# Supplementary material for: Effects of aerobic and resistance exercise on cardiac remodelling and skeletal muscle oxidative stress of infarcted rats
Source: J Cell Mol Med. 2020 Apr 2;24(9):5352–62. doi: 10.1111/jcmm.15191 (PMC7205792; doi:10.1111/jcmm.15191)
Supplement: Supplementary file 5 — Table S4 [file JCMM-24-5352-s005.docx]

**Supporting information 4.** Protein expression

|  | **Sham** | **S-IM** | **A-IM** | **R-IM** |
| --- | --- | --- | --- | --- |
| **20S Proteasome** | 1.00 ± 0.40 | 1.32 ± 0.31 | 1.05 ± 0.52 | 1.28 ± 0.59 |
| **Ubiquitin** | 0.49 (0.45-2.05) | 0.16 (0.13-0.62) | 0.58 (0.22-1.08) | 0.29 (0.11-1.23) |
| **Pax-7** | 1.00 ± 0.95 | 0.79 ± 0.36 | 1.31 ± 0.69 | 0.64 ± 0.48 |
| **Protein oxidation** | 1.00 ± 0.41 | 0.69 ± 0.33 | 0.46 ± 0.14 | 0.55 ± 0.36 |

S-MI: sedentary myocardial infarction (MI); A-MI: aerobic exercised MI; R-MI: resistance exercised MI; n=7 animals per group. ANOVA and Student-Newman-Keuls or Kruskal-Wallis; p>0.05.
